# Perforated PUD associated with pneumatosis intestinalis: A case report

**DOI:** 10.1016/j.ijscr.2025.111891

**Published:** 2025-09-02

**Authors:** Dereje Gebisa Bedada, Tafese Gudissa Merga, Alemu Bedado Hirpho, Daba Iticha, Kassahun Girma Desta

**Affiliations:** Department of Surgery, College of Health Science, Salale University, Fiche, Ethiopia

## Abstract

**Introduction and Importance:**

Perforated peptic ulcer disease (PUD) represents a serious complication of PUD. Its association with pneumatosis intestinalis (PI) is exceedingly rare. PI is identified by the presence of gas within the bowel wall. The clinical presentation and management of PI are contingent upon the underlying causes.

**Case presentation:**

We present the case of a 45-year-old male patient who arrived with a one-day history of abdominal pain. His vital signs were stable upon examination. During the abdominal assessment, findings included guarding and rigidity throughout the abdomen. The patient underwent laparotomy, which revealed a perforation in the first part of the duodenum and multiple air-filled cystic lesions on the small bowel. A duodenal repair, along with resection and anastomosis of the small bowel, was performed. The patient experienced a smooth postoperative recovery and has shown positive progress at the one-year follow-up.

**Clinical discussion:**

Perforated peptic ulcer disease (PUD) is a frequent reason for surgical admission. It typically presents as acute abdominal pain; however, its association with pneumatosis intestinalis (PI) is quite rare. PI, characterized by the presence of gas in the bowel wall, is an uncommon condition that can be classified as either primary (idiopathic), which occurs without any identifiable underlying condition or secondary, which occurs as a result of another medical condition. Diagnosis may be confirmed through imaging or during surgery, and histological evaluation is rarely necessary. The clinical presentation of PI can vary significantly. Management strategies depend on the classification of the condition: surgery is seldom required for idiopathic or benign secondary PI, whereas more severe cases of secondary PI usually necessitate surgical intervention.

**Conclusion:**

Although rare, pneumatosis intestinalis can occur in association with perforated peptic ulcer disease (PUD). Managing the underlying cause of the secondary pneumatosis does not require additional treatment.

## Highlights

• Perforated PUD is one of the serious complications of PUD.
• Perforated PUD association with pneumatosis is exceedingly rare.
• Pneumatosis intestanalis can be categorized as primary or secondary pneumatosis intestinalis.
• Clinical presentation and management of pneumatosis intestinalis depends on underlying causes.

## 1 Introduction

Perforated peptic ulcer disease (PUD) is one of the most severe complications associated with PUD. It typically presents with symptoms of acute peritonitis and is infrequently related to pneumatosis intestinalis (PI) (1,2). PI refers to the accumulation of gas within the intestinal wall. The exact pathophysiology of PI remains unclear, but it is thought to result from factors such as bacterial fermentation, mucosal disruption, and increased intraluminal pressure (3). PI can be classified as either primary (idiopathic) or secondary (4). The clinical presentation of PI varies depending on its underlying causes. Diagnosis may be achieved through imaging techniques or during intraoperative assessment via laparotomy or laparoscopy. Management strategies are determined by the specific underlying causes (5). Primary PI and benign secondary PI are generally associated with favorable outcomes, whereas severe secondary PI carries a high risk of mortality unless recognized and treated promptly (3,6). We present a rare case of perforated PUD associated with PI, which provides valuable insight into the complex mechanical etiology of PI. This case report has been reported in line with the SCARE checklist (15).

## 2 Case presentation

A 45-year-old male patient who presented with a one-day history of abdominal pain. Initially, the pain was localized in the epigastric region but later became generalized throughout the abdomen. Associated symptoms included vomiting of ingested matter in three episodes with vomitus consisting of freshly eaten food particles with high-grade intermittent fever, and appetite loss. Before this episode, he had experienced vague, intermittent abdominal pain for the past ten years and had been treated multiple times for peptic ulcer disease (PUD). He reported no changes in bowel habits.

Upon evaluation, his vital signs were stable. Blood Pressure was 95/60 mmHg, Pulse rate was 64 beats per minute, Respiratory rate was 18, and Temperature was 36.8 °C. The abdomen was flat and did not move with respiration. There was guarding and rigidity throughout the abdomen.

**Fig. 1:**
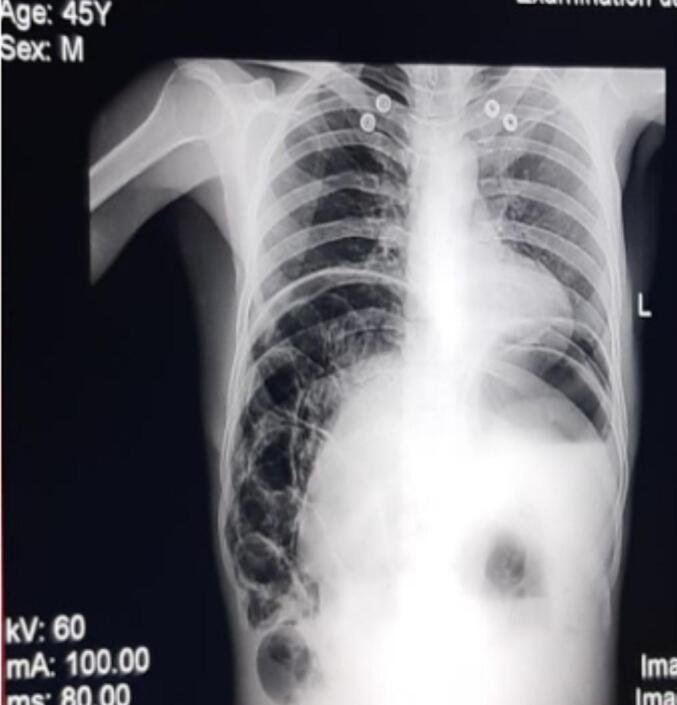
An abdominal x-ray shows free air under the right hemidiaphragm (blue arrow) and multiple cystic radiolucent areas within the bowel wall (white arrow).

A complete blood count (CBC) was performed and was largely within normal limits, except for neutrophilia at 78.8 %. Both the renal function test and liver function test were also normal. A plain abdominal X-ray revealed free air under the right hemidiaphragm and multiple cystic radiolucent areas within the bowel wall (Fig. 1).

**Fig. 2:**
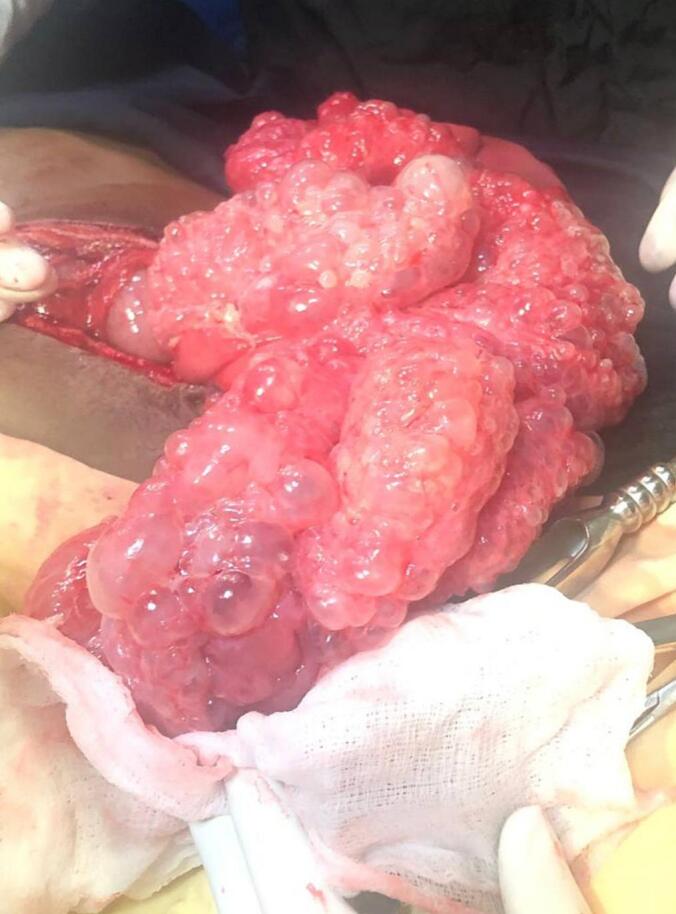
Multiple air-filled cystic changes are present in the bowel wall.

The patient was resuscitated with intravenous fluids and produced adequate urine output. Given the signs of an acute abdomen due to generalized peritonitis from viscus perforation, patient underwent laparotomy. During the operation, approximately 2000 ml of thin pus mixed with upper gastrointestinal (GI) contents were found within the general peritoneum. A perforation measuring 0.5 × 0.5 cm was identified on the anterior wall of the first part of the duodenum. Additionally, multiple air-filled cystic changes were observed 300 cm from the ligament of Treitz to 20 cm proximal to the ileocecal valve, totaling a length of 240 cm, along with multiple mesenteric lymphadenopathies (Fig. 2). Other organs were evaluated and appeared normal.

The GI contents and pus were sucked out, an omental patch was performed, the affected small bowel segment showing cystic changes was resected, and an end-to-end anastomosis was completed. The specimen was sent for histopathological evaluation, and the abdomen was lavaged with warm normal saline. The patient had a smooth postoperative course and was discharged on the 8th postoperative day. At the one-year follow-up, he reports having a smooth recovery with no recurrence of symptoms.

## 3 Discussion

Perforated peptic ulcer disease is a serious complication of peptic ulcer disease (PUD) characterized by a perforation in the stomach or duodenum. The pathological effects are typically localized to the site of the lesion. Patients with this condition often present with acute symptoms of peritonitis (2,7).

Pneumatosis intestinalis (PI) is characterized by the presence of gas within the wall of the intestines, which may occur in the submucosal or sub serosal layers (3). This condition can affect any segment of the gastrointestinal tract, from the esophagus to the rectum (8). The precise mechanism through which gas infiltrates the bowel wall remains unclear. It is believed to arise from factors such as mucosal breaches, bacterial transduction and gas production, as well as mechanical factors, including increased intraluminal pressure (4,8). In cases of perforated peptic ulcer disease (PUD), the occurrence of PI may be attributed to gas tracking from the ulcer through surrounding tissue planes (7).

PI can be classified into primary and secondary types. Primary PI has an unknown underlying cause and typically follows a benign course. In contrast, secondary PI is associated with various underlying conditions, including chronic lung disease (resulting from alveolar rupture), gastrointestinal disorders such as necrotizing enterocolitis, peptic ulcer disease, gastric outlet obstruction, mesenteric ischemia, lactulose intolerance, severe diarrhea, medication-induced gastrointestinal toxicity (4,9,10).

The clinical presentation of protein pneumatosis intestinalis based on its underlying causes. In cases of primary PI, patients may be asymptomatic or experience mild symptoms such as mild abdominal pain, diarrhea, bloating, excessive gas production, and mucoid discharge (11). Conversely, individuals with secondary PI typically exhibit symptoms related to the underlying condition. This group often presents acutely ill, experiencing severe symptoms, including abdominal distention, intense abdominal pain, vomiting, bloody diarrhea, and signs of systemic infection, with or without hemodynamic instability (12,13).

Differentiating between primary and secondary pneumatosis intestinalis (PI) is essential, as a delay in addressing secondary PI can be life-threatening for the patient. Diagnosis of PI can be achieved through imaging, endoscopy, or intraoperative inspection during laparotomy (6). Computed tomography (CT) scans are the preferred imaging modality for diagnosing PI (3). On a CT scan, PI typically presents as a linear or cystic hypo lucent area of gas collection within the bowel wall, which may be associated with pneumoperitoneum or portal venous gas in severe cases. Plain abdominal X-rays can also reveal similar findings, although their sensitivity is lower compared to CT scans (6). Abdominal ultrasound can be beneficial for diagnosing PI as well. During endoscopy, PI appears as a filling defect, which may be mistaken for a polyp; however, unlike a polyp, it collapses when subjected to bowel distention from intraluminal pressure (14). Caution should be exercised with endoscopy in the acute phase of the disease, as it poses a risk of intestinal perforation. Complete blood count (CBC) and renal and liver function tests, along with serum lactate levels, are valuable in assessing disease severity (6).

Management of PI is contingent on the underlying cause and the presence or absence of complications (3,6). Primary pneumatosis intestinalis often resolves with conservative treatment (9). Secondary PI caused by benign conditions, such as enterocolitis and severe diarrhea, can also be managed conservatively through methods like bowel rest, oxygen therapy, and antibiotics (5). Surgical intervention, including bowel resection and/or perforation repair, becomes necessary for patients experiencing bowel ischemia, intestinal perforation, or sepsis (3). The prognosis for PI varies significantly; those with primary and benign causes typically have favorable outcomes, while patients with secondary severe causes, such as intestinal perforation and ischemia, face a high mortality risk if not treated promptly (3,5).

## 4 Conclusion

Although it is rare to find peptic ulcer perforation-induced pneumonitis intestinalis, they can occur. Managing the underlying cause of the secondary PI does not require additional treatment.

## Author contribution

All authors contributed to different aspects. Dereje Gebisa operated on the patient, wrote the final case report and also following the patient. Tafese Gudissa wrote the case presentation and wrote the draft of the case report. Alemu Bedado, Daba Iticha and Kassahun Girma read and approved the final manuscript.

## Consent

A written informed consent was obtained from the patient for the publication of the case details and any accompanying images. A copy of the written consent is available for review by the Editor-in-Chief of this journal upon request.

## Ethical approval

Ethical approval is considered unnecessary by the Institutional Review Board since this is a single rare case encountered in clinical practice.

## Guarantor

Dereje Gebisa Bedada.

## Registration of research studies

Not applicable.

## Sources of funding

This manuscript was developed without the support of any specific grants from funding organizations in the public, private, or non-profit sectors.

## Declaration of competing interest

All authors have confirmed the absence of any potential conflicts of interest, ensuring transparency and integrity in their work.
